# Primary Chest Wall Ewing Sarcoma: Treatment and Long-Term Results

**DOI:** 10.3390/life14060766

**Published:** 2024-06-17

**Authors:** Ottavia Salimbene, Domenico Viggiano, Francesco Muratori, Roberto Lo Piccolo, Flavio Facchini, Angela Tamburini, Domenico Andrea Campanacci, Luca Voltolini, Alessandro Gonfiotti

**Affiliations:** 1 Division of Thoracic Surgery, Careggi University Hospital, 50134 Florence, Italyviggianod@aou-careggi.toscana.it (D.V.); luca.voltolini@unifi.it (L.V.); 2 Division of Oncological Orthopedics, Careggi University Hospital, 50134 Florence, Italy; muratorif@aou-careggi.toscana.it (F.M.); domenicoandrea.campanacci@unifi.it (D.A.C.); 3 Division of Pediatric Surgery, Meyer University Hospital, 50139 Florence, Italy; roberto.lopiccolo@meyer.it (R.L.P.); flavio.facchini@meyer.it (F.F.); 4 Division of Pediatric Oncology, Meyer University Hospital, 50139 Florence, Italy; angela.tamburini@meyer.it

**Keywords:** Ewing sarcoma, chest wall reconstruction, multidisciplinary approach

## Abstract

Objective: The aim of the study is to evaluate early and long-term results of chest wall primary Ewing’s sarcoma patients treated in the time period February 2000–February 2023 by a multidisciplinary approach. Methods: We retrospectively reviewed the medical records of patients who underwent chest wall resection for a primary tumor. Treatment approach, extent of resection, 30-day mortality, overall survival (OS), local recurrence-free survival (LRFS), and metastasis-free survival (MFS) were analyzed. Results: Overall, n = 15 consecutive patients were treated for chest wall primary Ewing’s sarcoma. A median of n = 3 ribs was resected with a median of n = 2 ribs adjacent to the lesion. Resections were extended to the adjacent structures in n = 5 patients (33.3%). In all cases, we performed a prosthetic reconstruction, associated with muscle flap (n = 10, 66.6%) or with rigid titanium bars and muscle flap (n = 6, 40%). A radical resection was accomplished in n = 13 patients (84.6%). The median surgical time was 310 ± 120 min; median hospitalization was 7.8 ± 1.9 days. Post-operative mortality was zero. We recorded n = 4 (30.7%) post-operative complication. The median follow-up (FU) was 26 months. Moreover, 5-year overall and event-free survival were 52% and 48%, respectively. Conclusions: This case series confirms the benefit of the multidisciplinary approach for Ewing sarcomas in early and long-term results.

## 1. Introduction

Within the great family of the chest wall tumors (CWTs), in children, we can find both malignant and benign neoplasms. Among the first, Ewing’s sarcoma (ES), historically known as Askin tumors, is the second most common primary malignant bone cancer. Although it can manifest at any age, is the most frequent chest wall tumor in children and young adults [1], with a median age of occurrence between 16 and 25 years [2,3] and a prevalence in the male sex. This tumor is considered to be derived from a mesenchymal progenitor cell [4] represented by neuroectoderm and undifferentiated neuroepithelial cells and origins from the bone components of the chest wall (9% ribs, 4% scapula, 1% clavicle, <1% sternum) [2]. All ES are characterized by the presence of non-random chromosomal translocations producing fusion genes that encode aberrant transcription factors, whose targets are oncogenes, oncosuppressor genes, and genes related to apoptosis, differentiation, angiogenesis, and invasion [5,6]. The t (11; 22) (q24; q12) translocation appears in approximately 85–90% of ES [7] and is about 90% specific [8,9]. Since ES is an extremely aggressive tumor, it should be considered a systemic disease at presentation because of the high frequency of metastatic spread and local recurrence and should be treated using a multimodal approach (surgery, radiotherapy, chemotherapy). Moreover, given its rarity and the complexity of management, bone sarcoma should be treated in reference centers and/or within reference networks able to provide access to the full spectrum of care and age-specific expertise [10].

There is no well-defined and standardized approach for chest wall ES. The main reason why there is no uniform approach is the low incidence of these malignancies, which has not allowed to define an unambiguous treatment modality, especially regarding local control. Consequently, the treatment of ESFTs is complex and relies on a multidisciplinary approach. The treatment for ESFTs includes local control of the disease—including surgery, radiotherapy, or a combination of both—and systemic therapy, based on chemotherapy in neoadjuvant and/or adjuvant regimens [11,12,13]. This multimodality approach leads to a 5-year overall survival of 60–70% [14,15,16]. Patients who undergo surgical resection are a small group, and there is a lack of publications regarding this specific topic.

Our purpose is to share our experience of 15 patients affected by an uncommon cancer describing the treatment and the post-operative outcomes.

## 2. Materials and Methods

We retrospectively reviewed the medical record of all patients who underwent CW resection for primary Ewing’s sarcoma treated at University of Florence, Italy, during a 22-year period. Most of our operations were planned and performed by a team composed of a thoracic surgeon, an orthopedic oncologist, and a plastic surgeon. With the aim of studying the soft tissue involvement, all patients underwent chest Magnetic Resonance Imaging (MRI), a total-body Computed Tomography scan (CT), Bone Scintigraphy until 2010, and Positron Emission Tomography (PET) after 2010. Based on imaging, patients were graded according to the Enneking classification (Muscle-Skeletal Tumor Society) and according to the classification of the American Joint Committee on Cancer Staging System (AJCC). A complete work-up was undertaken in all cases to eliminate a disseminated disease. Where the histological diagnosis was not present at the time of our observation, we performed a core-needle aspiration or an excisional biopsy; fine-needle aspiration was not included in our diagnostic work-up. Patients were all evaluated during the weekly meeting of the Institutional Sarcoma Group, comprising an orthopedic oncologist, thoracic surgeon, oncologist, radiotherapist, radiologist, pathologist, and pediatric oncologist. All treatment protocols used for ES consisted of neoadjuvant treatment and surgery plus adjuvant treatment with or without radiotherapy [17]. After induction chemotherapy, patients underwent surgical resection and CW reconstruction as a one-stage procedure and following a specific surgical policy. The site of the biopsy was always included in the skin incision; the extent of resection concerned the lesioned ribs, the adjacent intercostal space, and ribs with 4 cm free margin proximally and distally to the tumor, though an en-bloc resection. Lungs and/or other organs (pleura, pericardium, thymus, diaphragm) were involved in the resection when needed, as well as the previous irradiated skin if RT was performed. According to the Enneking classification [18], adapted by the Scandinavian sarcoma pathology group for the chest wall [19], we classified the surgical margins into three types: type I: wide (pleura internally and muscle fascia externally intact with >2 cm macroscopic and microscopic free from tumor); type II: marginal (<2 cm margin free from tumor); type III: intralesional (lesion taken out in pieces or microscopic positive margins). The Enneking classification includes “radical” surgical margins as well, achieved through a radical amputation; we cannot include type IV in our study because it cannot be applied in chest wall surgery. Pathological specimens were analyzed by expert pathologists, and Ewing sarcoma was classified from grade 1 to 3 [19,20]. The aim of the reconstruction of the chest wall after oncological resection is to restore the thorax structural stability and functional integrity, protecting the underlying organs and preventing lung herniation with aesthetically acceptable results. To meet these outcomes, we used soft tissue coverage and prosthetic materials (both rigid and non-rigid). Nowadays, there are no guidelines about the indication and the modality of chest wall reconstruction although many surgeons agree on a few points: the rigid reconstruction should be performed on >5 cm chest wall defect after 3 ribs’ removal from the anterior chest wall and 4 ribs’ removal from the posterior chest wall. The extent of the resection should be planned before surgery and, of course, during the operation, involve other professionals (e.g., plastic surgeon). After surgery, all patients were immediately extubated and transferred to the Intensive Care Unit (ICU). Since the immediate post-operative period, patients were followed by professionals for respiratory physiotherapy. About an adjuvant treatment, the need to perform induction chemotherapy or post-operative chemotherapy and radiotherapy was discussed and planned with the medical oncologist and the radiotherapist in an oncological multidisciplinary group. Survival was calculated from the date of surgery to death or date of last follow-up for those patients who survived and estimated by the Kaplan–Meier product-limit method.

Statistical analysis was performed using SPSS version 24 (IBM Corporation, Armonk, NY, USA). The Kaplan–Meier method was used to calculate the OS, LEVS, and MFS. The results of the analyses are expressed as odds ratios (ORs), and *p*-values below 0.05 were considered statistically significant.

## 3. Results

In the selected period, n = 15 consecutive patients (9 males, 60.0% and 6 female, 40.0%) were treated at the University of Florence Sarcoma Group because they were affected by a primary CW Ewing’s sarcoma. There were n = 14 first procedures and n = 1 local relapse after upfront surgery performed elsewhere. All patients underwent a multimodality treatment with surgical resection of the tumor. The median age at onset was 23.6 years (range: 2–61). The most salient data of each patient are included in Table 1.

The first symptoms in all patients were pain in the affected region, swelling, or both; some patients also presented dyspnea, asthenia, cough, and weight loss. The thoracic wall represented the starting point of development for all the lesions which were fast growing.

In regard to the stage, following the Enneking classification, n = 5 cases were classified as stage IIA and n = 10 cases as stage IIB, while according to the AJCC system, n = 7 cases were stage IIA, and n = 8 cases were stage IIB.

Before surgery, all patients had been biopsied with histological and immunological examination; using the Reverse Transcription Polymerase Chain Reaction (RT-PCR) technique, we identified the ESFTs typical translocation t (11; 22), and t (21; 22): n = 12 cases were positive for 22q12 (EWSR1). In n = 2 cases EWSR1, was absent, and in n = 1 case, it was not assessable.

An induction chemotherapy was performed in all patients: n = 7 cases were treated according to the Italian Sarcoma Group (ISG) and Scandinavian Sarcoma Group (SSG) trial ISG/SSG III [21], n = 3 cases according to the ISG and the Italian Association of Pediatric Hematology and Oncology (AIEOP) trial ISG/AIEOP EW-1 [22], n = 4 cases according to the ISG and the AIEOP trial ISG/AIEOP EW2 [23], and n = 1 case according to the ISG/AIEOP EW Oss protocol.

The characteristics of resections are illustrated in Table 2. In all cases, we resected the lesion with wide margins: a median of three ribs were resected (range: 3–7) with a median of two ribs adjacent to the lesion (range: 1–4). We needed to expand the resection at the adjacent structures in six patients (40%): in n = 3 cases (20%), we performed a lung wedge resection; in n = 1 (6.6%) case, a left pleuro-pericardial pneumonectomy; and in n = 2 (13%) cases, a partial diaphragm resection.

All chest wall resections underwent reconstruction for both cosmetic and functional reasons. The types of reconstructions are resumed in Table 2.

We performed a prosthetic reconstruction using an expanded polytetrafluoroethylene (ePTFE) 2 mm thick patch (Dual Mesh plus, W.L. Gore & Assoc, Flagstaff, AZ, USA) in the first n = 4 cases; since 2012, we have used a porcine dermal collagen matrix that is 1.5 mm thick (Permacol, Medtronic, Dublin, Ireland) as a biological mesh (n = 11 patients). Reconstruction was associated with muscle flap in n = 10 (66.6%) patients; muscle flaps were: unilateral latissimus dorsi (n = 5), bilateral latissimus dorsi (n = 1), and pectoralis major (n = 4). A combined titanium rigid (Strasbourg Thoracic Osteosynthesis System (STRATOS) MedXpert GmbH) and non-rigid prosthetic reconstruction together with muscle flap was performed in n = 6 (40%) patients. Rigid reconstruction was always added in the case of antero-lateral chest wall resections.

About surgical margins, type I margin was accomplished in n = 13 cases (86.6%), type II in n = 2 cases (13.3%), and no cases resulted in type III. Histological examination of ES demonstrated G1 tumor in three (20%) patients, G2 in eight (53.3%), and G3 in four (26.6%) patients. The median surgical time was 310 ± 120; median patient hospital stay was 7.8 ± 1.9 days. Post-operative mortality was zero. The morbidity rate was 20%: n = 1 patient developed atrial fibrillation occurred with no hemodynamic instability, successfully treated by pharmacological cardioversion (intravenous amiodarone); n = 1 required four blood transfusions (two during the surgical time and two after surgery) for anemia without obvious sources of active bleeding, probably due to the extent of the surgical removal (pneumonectomy and resection of the II-III-IV-V ribs); n = 1 patient developed a wound infection suffering with necrotic area. After the removal of the necrotic portion and a curettage, the resitutio ad integrum occurred thanks to the Vacuum-Assisted Closure (VAC) therapy kept in place for two weeks replacing it every 48 h. Other minor (n = 2) seromas were treated conservatively without the use of drainages, but through two liquid aspirations in one case and three aspirations in the other and a compressing medication with a resolution in 13 days and 17 days, respectively (Table 3). All patients underwent adjuvant chemotherapy. Additional post-operative irradiation was administered in two cases due to the above-mentioned marginal resection.

**Figure 1:**
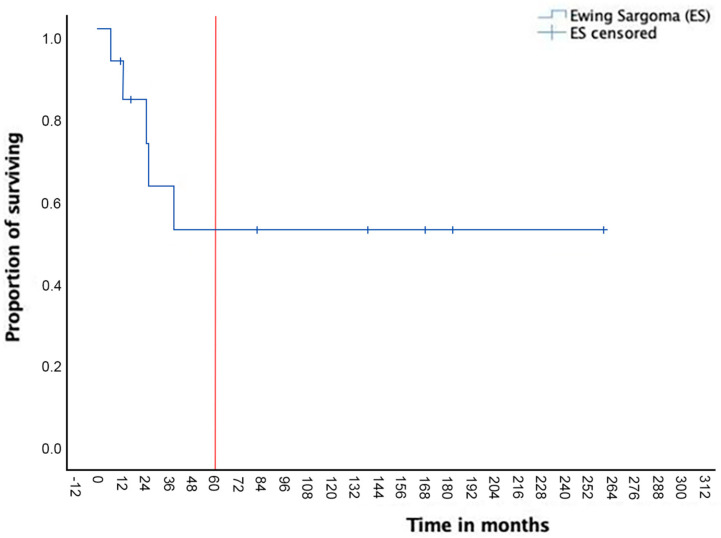
The 5-year overall survival (OS).

**Figure 2:**
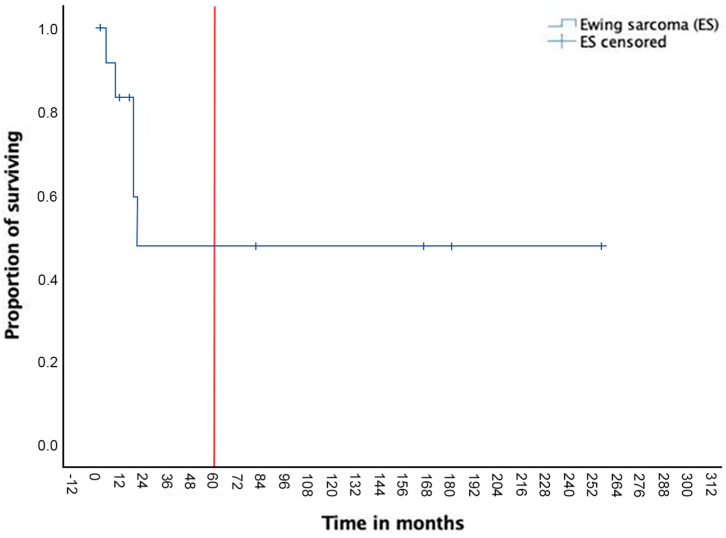
The 5-year event-free survival (EFS).

The median FU was 26 months (mean: 69, range: 7–256). A re-intervention was necessary in two patients because of local recurrence, occurring, respectively, at 13 and 19 months after the primary resection. N = 1 redo surgery consisted of a new extended rib resection (n = 3 ribs) plus partial diaphragm resection. N = 1 CW resection (n = 2 ribs plus partial sternectomy) was extended to the diaphragm and to the parietal pleura (partial pleurectomy). In two other patients, local recurrence occurred, although surgery was not indicated also because of the presence distance metastasis. Another patient underwent lung resection for multiple metastasis 28 months after the chest wall resection and died after 6 months because of brain metastasis. The 5-year OS was 52% (Figure 1); EFS at 5 years was 48% (Figure 2).

## 4. Discussion

Primary malignant CWTs are rare, accounting for less than 1% of all primary tumors [24]; among these, ES is an extremely aggressive tumor with frequent metastatic spread and local recurrence. It should be considered to be a systemic disease at presentation [25] with a multidisciplinary management; in this contest, surgery is the cornerstone of treatment in order to obtain local control, as large studies have already assessed [26]. The peak incidence of Ewing’s sarcoma is 13–16 years, with a male:female ratio of 2:1. Our series confirms these data, with a wide male prevalence (61.5% vs. 38.5); furthermore, our pediatric population confirms the interval of 13–15 years as the peak incidence timeframe. The first presenting symptom is usually an increasing pain that can be associated with cough, pain fever, malaise, anemia, and a high erythrocyte sedimentation rate. In our series, pain and swelling were the most frequent symptoms. Just one patient came to our attention because of respiratory symptoms, (dyspnea, cough, and hypoxia), which is an uncommon presentation setting. This clinical scenario was due to a giant lesion occupying the entire left hemothorax, compressing and dislocating the mediastinum because of an internal growth prevalent over the external one (no swelling was observed), causing a very delayed diagnosis. As M. Rocca et al. underlined, to perform an adequate surgery, a correct preoperative staging is mandatory; CT or MRI are the best techniques to conduct preoperative planning, directed towards tumor characterization and definition of its extent [27]. In our series, both pediatric and adult patients performed a pre-op staging by CT and MRI to better pre-operatively assess the extent of resection and the need of reconstruction. CT and MRI were performed before and after induction chemotherapy to evaluate treatment response; however, the extent of resection was always planned considering the pre-treatment radiological images. In our mind, this policy should better achieve free surgical margins. Sedaghatl et al. demonstrated the importance of MRI to detect local recurrence during the FU, with the advantage of high soft-tissue contrast and no radiation; in their study, all of the patients were examined using contrast-enhanced MRIs, which showed high sensitivity and specificity (92% and 98%, respectively) [28]. Similarly, in another study, the authors showed the value of MRI in detecting the occurrence after resection of primary STS (subcutaneous edema, muscle edema, and seroma) [29].

The main issue around surgery concerns the timing between CT and surgery: What is better, to perform surgery first and then CT, or to administer a neoadjuvant CT followed by excision of the tumor? Our Sarcoma Group opted for the second choice, CT + surgery. It showed, in fact, excellent results in reducing the tumor’s size and, even if not allowing less extensive chest wall resections, we speculate that reduction in neoplasm volume gave us the possibility to reach a high rate of wide margin resections, probably reducing microscopic margin infiltration. Seits et al. noticed that with neoadjuvant chemotherapy, there was a more frequent complete tumor resection [30]. Shamberger et al. [31] studied the impact of initial versus delayed resection on tumor margins and survival in CW Ewing’s sarcoma, having noticed that 10 out of 20 (50%) initial resections resulted in negative margins compared with 41 out of 53 (77%) negative margins of patients undergoing chemotherapy first, followed by surgery (*p* = 0.043). As a Scandinavian Sarcoma Group study reported, surgery has, as a main objective, achieving disease-free margins (R0) [19] through an aggressive bone and soft tissues resection. Bedetti et al. also showed that the best way to achieve a local control is through a microscopically complete tumor resection [2]. It is still a matter of discussion as to whether the addition of RT benefits survival as opposed to surgery alone. D.J. Indelicato et al. reviewed the 40-year University of Florida experience treating ES: local control was not statistically significantly different between patients treated with RT alone (61%) vs. surgery and RT (75%) [32]. ES are therefore undoubtedly radiosensitive tumors; nevertheless, in our center, we tend to use radiation only in in patients with incomplete resection [2] (in n = 2 patients with surgical margin type II) to avoid the risk of complications associated with radiation.

OS was 78 and 71% at 3 and 5 years, respectively, in the study of Bedetti, while EFS was 71 and 65% at 3 and 5 years, respectively [2], in line with Basharkhah et al. who found a 5-year OS of 71% in seven patients [20]. In a review of 104 patients, Laskar et al. found an OS of 54% and a EFS of 36% [33]. Our results are in line with this latest study; in our series, we found an OS of 52%.

Provost et al. [3] found that the presence of pleural involvement or effusion was associated with worse survival outcomes, similar to those reported in metastatic Ewing’s sarcoma. These results support those of Lin and colleagues [34] and Laskar et al. [33], regarding pleural involvement as an important prognostic factor. We also observed cases with pleura and lung resulting in poor prognosis: six patients, showing pleural involvement at the time of relapse, died 9 months after relapse.

Laskar et al. showed how an <8 cm size of tumor has an OS of 25% despite a size > 8 cm sized tumor with an OS of 10% [33]. Even in our experience, we observed that the tumor’s volume at the onset, and therefore the number of ribs involved, is a negative prognostic factor; 85% of the recurrences occurred in patients with more than two ribs involved, with an OS of 25%. Unfortunately, due to a small number of patients, these data are not statistically significant. Another important factor with a statistically significant negative influence on OS and DFS is the age > 18 years [35]. Data analyzed by Cotteril et al. underlined the importance of age: in a series of 975 patients from the European intergroup cooperative Ewing’s sarcoma study group (EICESS), patients’ EFS < 15 years was 63%, while older patients’ EFS was 52% [36].

Saenz also reported age > 10 years was associated with a significantly worst outcome compared with ages < 10 years; even if the reason remains unclear, it should be caused by altered tumor biology in older children [37]. Our data show a similar trend, but since we analyzed a small patient series, we cannot exclude this correlation.

Very little literature exists about the recommended management strategies for pediatric surgical resection and reconstruction for CWTs. In this series, we followed the principle of different reconstruction based on the patient’s age and body growth. The C. Dingemann et al. study demonstrated that surgical reconstruction after the resection of malignant CWTs using non-rigid prosthetic material is safe and effective in pediatric patients [38]. Particularly, like N. Maistri et al. [39], we support the use of biological graft in pediatric chest wall reconstruction; consequently, we chose only biological material in the case of patients younger than 13 years. Thanks to the excellent biomechanical characteristics and histologic remodeling of biological meshes, these do not interfere with chest wall growth, and they do not act as a foreign body material, minimizing the risk of infection and mechanical stress to the chest wall during physiological growth. While synthetic tissue materials remain a foreign body, biological mesh represents an extracellular scaffold for a physiologic tissue growth up to an integration of the prosthetic component with the surrounding tissues. Synthetic meshes and titanium represent a solid reinforcement structure, but in our historical experience, these may case an infection which may, in some cases, result in a surgical redo for their removal.

Biological mesh can be derived from human (allograft; derived from dermis, intestinal mucosa, or pericardium) or animal (xenograft; usually porcine or bovine) tissues. We always used a porcine-derived acellular cross-linked dermal matrix (PACLIDEM), a collagen matrix submitted to a decellularization process to remove cells, cell debris, DNS, and RNA. With the intention of giving more stability and reducing collagenase’s degradation the acellular matrix is cross-linked with hexamethylene diisocynate; as a result, it increases the tensile strength of the tissue, which is one of the main objectives for chest wall reconstruction.

The integration of rigid prosthetic systems with biological meshes is intended to increase the strength of the reconstruction; however, it is burdened by an increased risk of infection [13,40,41].

Reconstruction with PACLIDEM alone has always been successful; among the four patients reconstructed with synthetic mesh, there was one complication of the muscle flap and one wound seroma. In addition, the anterior chest wall reconstructions have all also required the use of rigid reconstruction systems. Despite the complexity of the reconstructions using different prosthetic systems and myo-cutaneous flaps, we recorded complications in four patients (31%), which seems acceptable in relation to the challenge of the cases.

No patient needed prosthesis removal due to infection or for any other reasons.

Limitations of the study are the retrospectivity and the sample size, which did not allow further statistical analysis (e.g., multivariate analysis). However, the multidisciplinary management proposed in this study was conducted within an appropriate time range and by the same team, reducing possible bias.

Given the rarity of the pathology under study, two cases from this series are proposed as examples.

## 5. Case Report n.1

**Figure 3:**
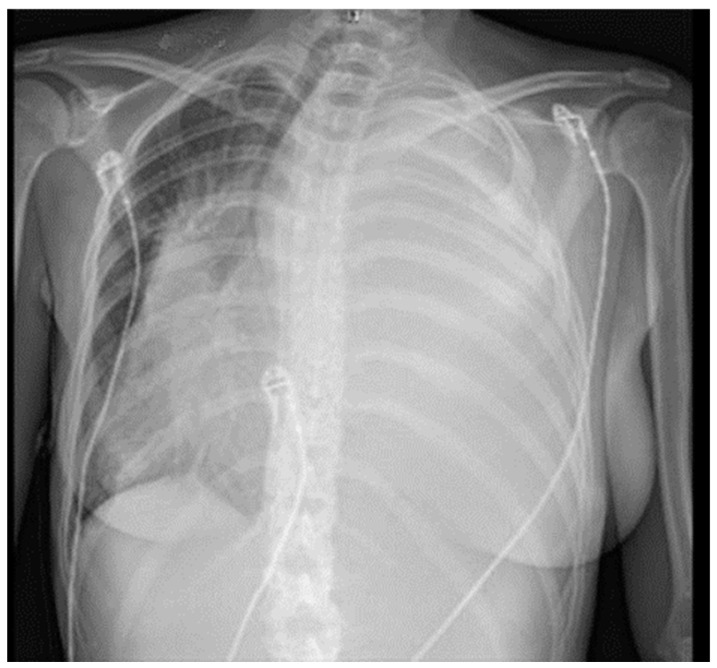
Chest X-ray at presentation.

A 19-year-old female was admitted to the ER because of significant weight loss, anemia (Hb 7 g/dL), fatigue, dyspnea, cough, tachycardia, and tachypnoea. The symptoms started four months prior to presentation, treated—in the meantime—with martial therapy and folic acid supplementation. Her family history was non-significant, as well as her clinical history and physiological anamnesis. The physical exam was remarkable for decreased breath sound of both the left lung upper and lower fields. No other pertinent positives were detected. Initial hematochemical analysis highlighted leukocytosis (WBC 16.6 × 10^9^/L), anemia (Hb 7 g/dL), and increased CRP (66 mg/L; normal values < 5–10 mg/L) and procalcitonin (0.85 ng/mL; normal values < 0.05 ng/mL). On EGA examination, the patient was hypoxemic. Conventional radiography in two planes was the first radiological investigation: chest X-ray showed complete radio-opacity of the left hemithorax, flaring of mediastinum on the right, compressed and laterally deviated trachea on the right, and a diffuse decrease in the normal density of the left III–IV ribs (Figure 3).

**Figure 4:**
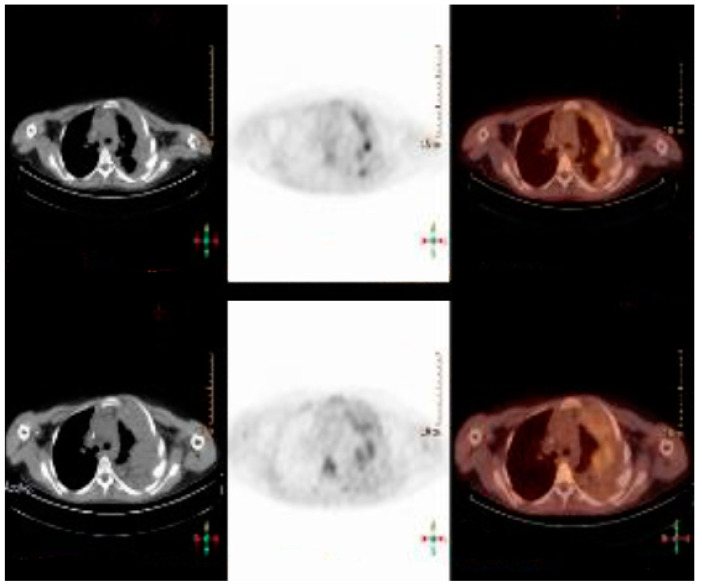
FDG-PET obtained after the fourth cycle of CT.

Based on the history, physical exam, and X-ray results, a thoracic-abdomen contrast CT was performed, which demonstrated a neoformation of 12 × 15 × 22 cm (AP × TR × CC) of the left lung, infiltrating homolateral muscle and ribs, with heart compression, subclavian vein dislocation, pleural and pericardial effusion. Then, US-guided biopsy of the chest wall lesion was carried out: on microscopy, a poorly differentiated malignant small round cell tumor was noted. The immunohistochemistry findings were consistent with Ewing’s sarcoma (gene EWSR1 translocation). The patient was immediately initiated on ISG/AIEOP EW-2 protocol, followed by local control through surgical excision, and then maintenance phase with oral cyclophosphamide and celecoxib. The treatment course was complicated by mucositis and ifosfamide neurotoxicity (myoclonias-like involuntary movements in all four limbs), for which prophylaxis therapy with methylene blue was carried out; in addition, admission for fever due to neutropenia was necessary. An MRI of the whole body was obtained after the first cycle of chemotherapy, which showed a significant decrease in the size of the tumor. The mass that was previously measuring 21 × 15 × 22 cm (AP × TR × CC) decreased to a size of 15 × 12 × 15 cm in its maximum dimensions. A chest MRI obtained after the fourth cycle of chemotherapy revealed a further reduction of the right thoracic mass (dimensions of 11 × 5 × 11 cm), with no alteration of the rib’s damage. A 18F-FDG-PET was obtained after the last cycle of neoadjuvant chemotherapy, which showed persistence of tissue with high glucose metabolism located at the known lesion at the level of the left hemithorax (Figure 4).

**Figure 5:**
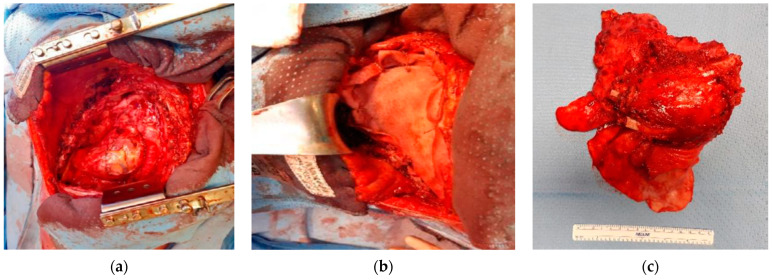
Images of the surgery: (**a**) surgical field after tumor resection; (**b**) chest wall reconstruction with non-rigid prosthesis; (**c**) specimen.

**Figure 6:**
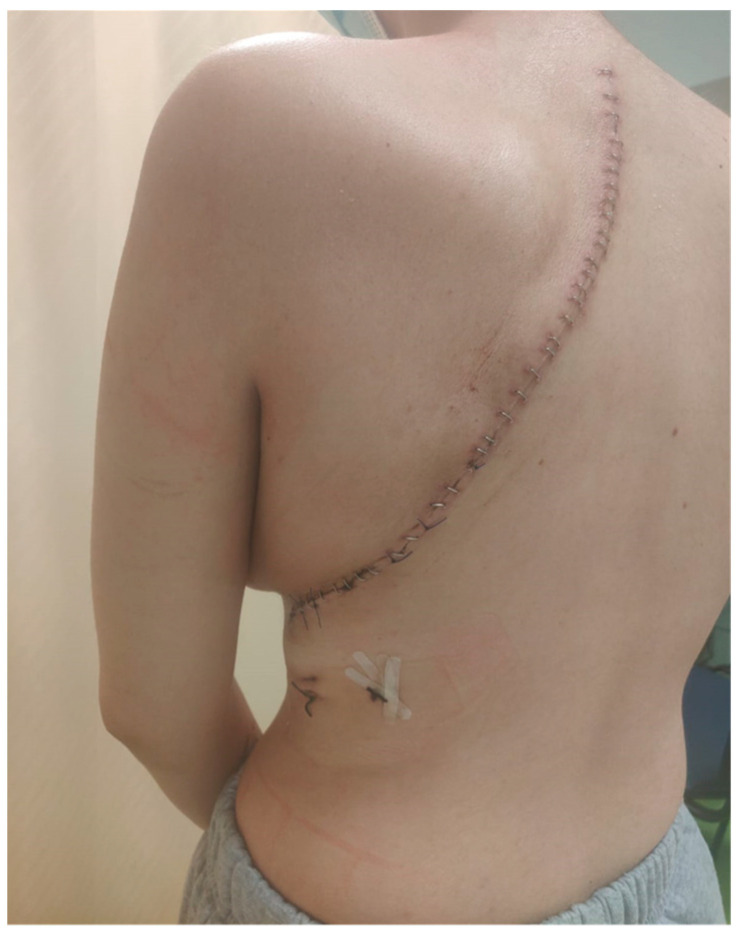
Aesthetic result.

The patient continued to receive the planned four cycles of induction chemotherapy; then, local control by surgical resection was decided. The patient underwent tumor wide resection—consisting of left pneumonectomy and resection of the II-III-IV-V ribs—and reconstruction with Permacol (Figure 5), obtaining an aesthetically pleasing result (Figure 6). The patient needed two blood transfusions during the surgery and two additional transfusions at the end, with 4 UI of plasma. No further complications were encountered during surgery.

**Figure 7:**
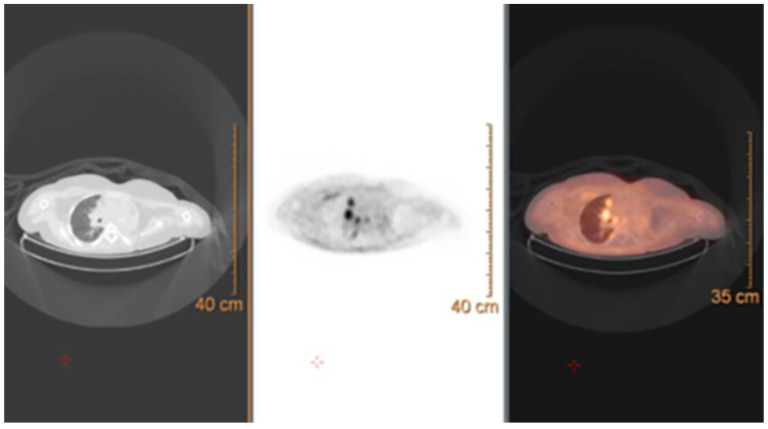
Post-operative FDG-PET.

Adjuvant chemotherapy based on cyclophosphamide and celecoxib was administered two months after surgery. The patient presented tachycardia and an elevation of cardiac enzymes; therefore, after cardiological counselling, a therapy with bisoprolol and ivabradine was initiated, with improvement of the cardiac function. Unfortunately, post- operative 18F-FDG-PET (Figure 7), performed 6 months after surgery, showed both local recurrence and diffuse metastasis (mediastinal and right thoracic wall hypercaptation), which led the patient’s death two months later.

## 6. Case Report n.2

**Figure 8:**
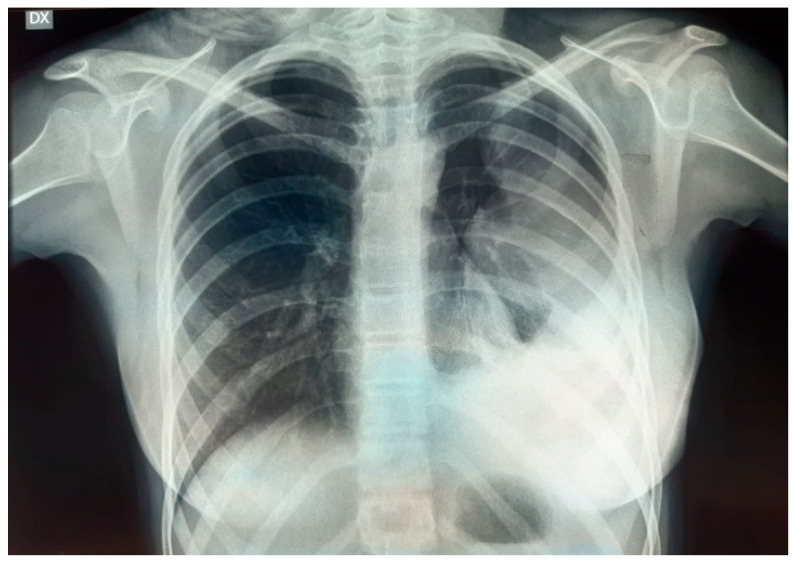
Chest X-ray at presentation.

A 15-year-old female was admitted to the ER for a voluminous asymmetric swelling of the left lateral chest wall, without a history of trauma or clinical signs of infection. The patient’s family and clinical history as well as her physiological anamnesis were non-significant. As the case above, diagnosis came through laboratory studies and instrumental investigations. In the hematochemical analysis we found out leukocytosis (WBC 14.9 × 10^9^/L) and no anemia but an increase of CRP and procalcitonine (59 mg/L and 0.70 ng/mL respectively). The chest X-ray demonstrated a significant radio-opacity of the left hemithorax at basal level (Figure 8).

**Figure 9:**
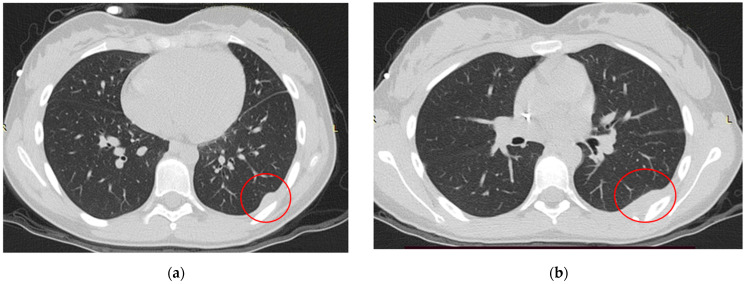
(**a**,**b**). TC scan after the second cycle of CHT.

The thoracic-abdomen CT showed two adjoining partly confluent solid neoformations of 4.3 × 1.9 × 3.8 cm and 2.4 × 1.4 × 3.1 cm attached to the left lateral rib pleura from the sixth rib to the seventh rib. The diagnosis was made by US-guided biopsy, which highlighted EWSR1 gene rearrangement, the Ewing sarcoma’s characteristic. The patient was subjected to ISG/AIEOP EW-2 protocol; the treatment course was complicated by an urticaria reaction following platelet transfusion. A TC scan (Figure 9) performed after the second cycle of chemotherapy showed a decrease in tumor size; the two lesions decreased to a size of 3.6 × 1.4 × 3.4 cm and 2.4 × 1.1 × 2.4 cm, respectively.

The operation consisted of a wedge pulmonary resection en bloc with the postero-lateral arch from the third to the ninth ribs, followed by the reconstruction with non-rigid (Permacol) and rigid prothesis. No post-operative complications occurred.

**Figure 10:**
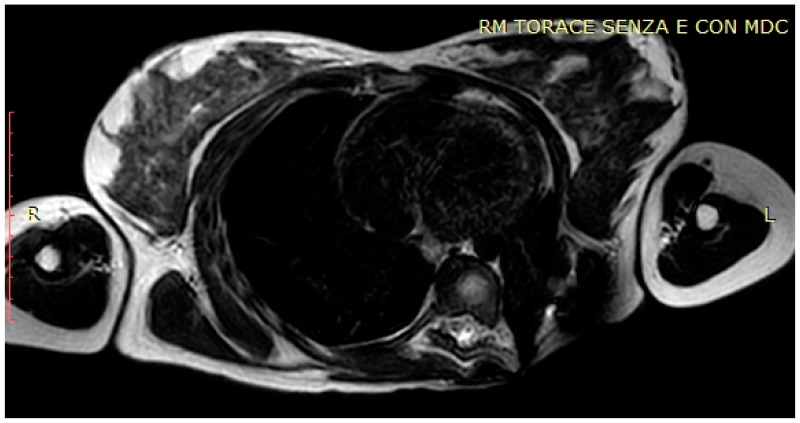
MRI after surgery.

An MRI (Figure 10) and a 18F-FDG-PET showed the absence of the heteroplastic tissue after surgery.

The surgery was followed by four cycles of maintenance chemotherapy. At the last follow up control, at 9 years after the diagnosis, there was no evidence of disease.

## 7. Case Report n.3

**Figure 11:**
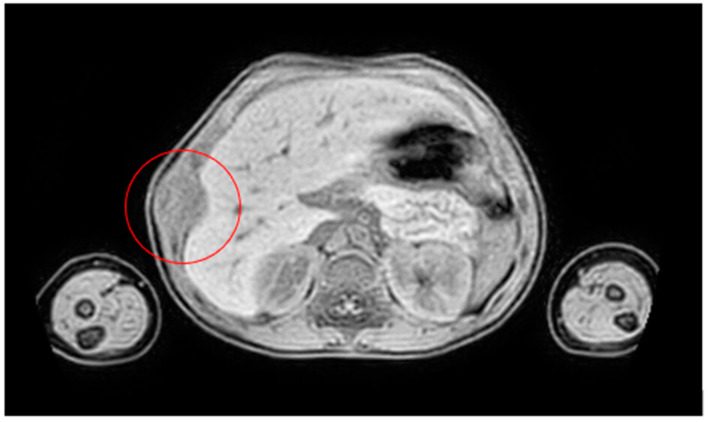
MRI at the presentation.

A 2−year−old female presented a swelling of the anterior arch of the ninth right rib, with no pain associated. In the blood tests, no alterations were found. The ultrasound exam showed a cartilaginous lesion of the ninth right rib, measuring 12 × 20 mm, confirmed by the MRI (Figure 11).

**Figure 12:**
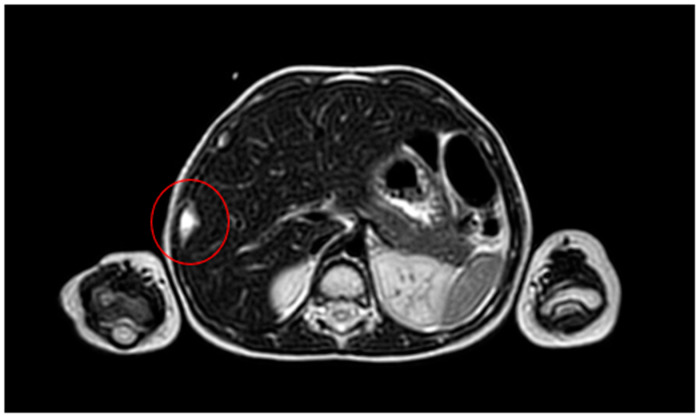
MRI at the end of the adjuvant CHT.

A US-guided biopsy was carried out; the immunohistochemistry findings were consistent with Ewing’s sarcoma (gene EWSR1 translocation). The patient was subjected to the ISG/AIEOP EW-1 protocol; the adjuvant CHT was complicated by vomiting and diarrhea with a C. Difficile positivity, fever, and neutropenia were treated with antibiotics and parenteral nutrition. Moreover, before the last cycle of adjuvant CHT, a G-CSF stimulation was performed to collect CSE in prevision of medullary aplasia. The chest MRI (Figure 12) obtained at the end of the neoadjuvant CHT showed the persistence of the ninth rib lesion.

**Figure 13:**
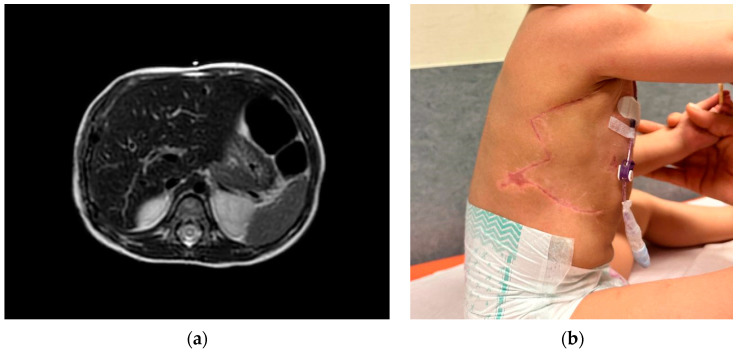
Post-operative results: (**a**) MRI after surgery; (**b**) aesthetic result.

The operation consisted of a wide resection en-bloc of the 8th–9th–10th ribs with a portion of diaphragm, reconstructed through a non-rigid prosthesis only. Three weeks later, a wound dehiscence required a VAC system for three weeks (changed every 48 h). The chest MRI performed after surgery presented no evidence of disease, and a very aesthetically pleasing result was obtained (Figure 13).

The adjuvant CHT was complicated by fever and oral mucositis treated efficiently with antibiotics.

## 8. Case Report n.4

**Figure 14:**
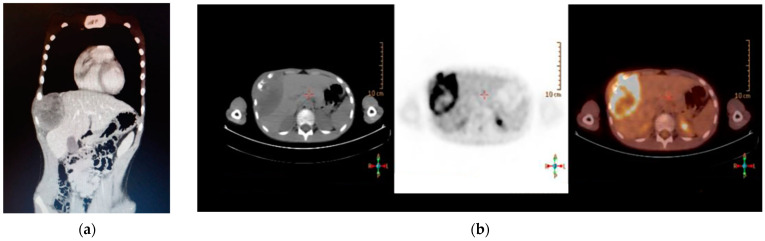
Images at presentation. (**a**) CT scan; (**b**) PET scan.

A 14-year-old male was admitted to the ER for a voluminous asymmetric swelling of the right lateral chest wall, without history of trauma or clinical signs of infection. The patient’s family history was non-significant nor were his clinical history and physiological anamnesis. The TC scan showed a thoracic wall mass measuring 10 × 7 × 8 cm, involving the 7th–8th–9th, right ribs, hypercapant at the 18F-FDG-PET exam (Figure 14).

**Figure 15:**
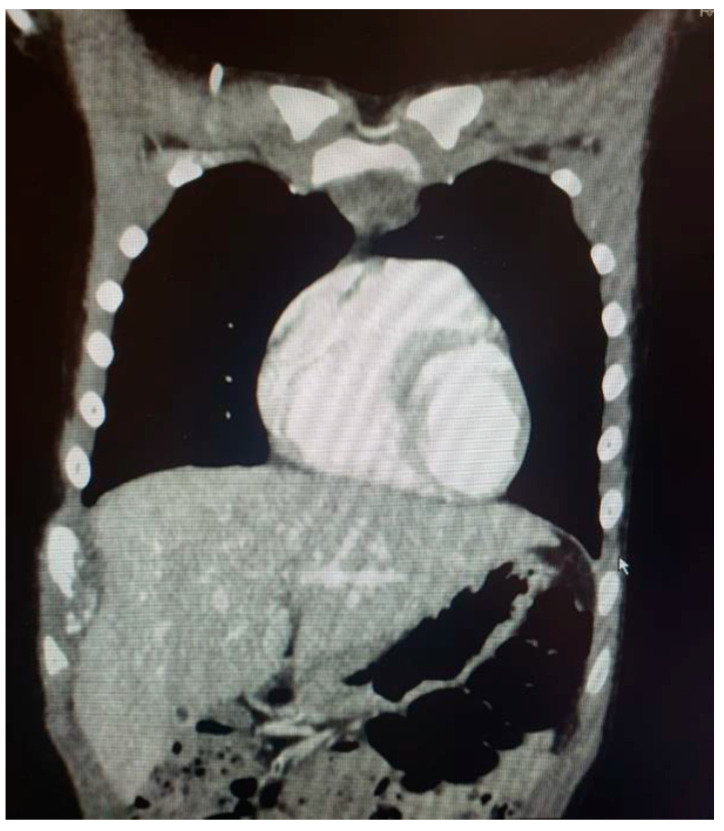
Post-induction CT.

The diagnosis was made by US-guided biopsy, which highlighted EWSR1 gene rearrangement. The patient was initiated on ISG/AIEOP EW-2 protocol, complicated by admission for fever due to neutropenia, following the first and the second cycle. An MRI of the whole body performed after the second cycle of chemotherapy showed a significant decrease to a size of 5.3 × 3 × 7 cm. A contrast CT of the whole body carried out 40 days later showed a further reduction in the size of the expansive formation (2.5 × 4.2 × 5.6 cm) (Figure 15).

**Figure 16:**
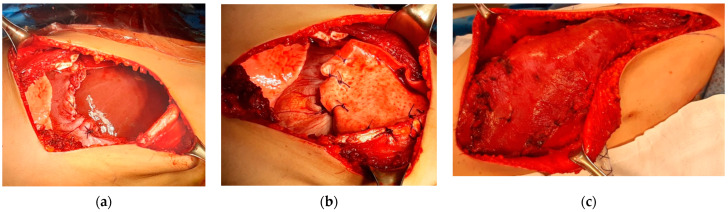
Operative management: (**a**) surgical field after tumor resection; (**b**) chest wall reconstruction with non-rigid prosthesis; (**c**) chest wall reconstruction with latissimus dorsi muscular flap.

The patient underwent radical surgery with a wide resection including the entire lesion and the complete removal of the 7th–8th–9th–10th ribs. The chest wall and diaphragm reconstruction were obtained by the application of Permacol and a flap with latissimus dorsi (Figure 16).

**Figure 17:**
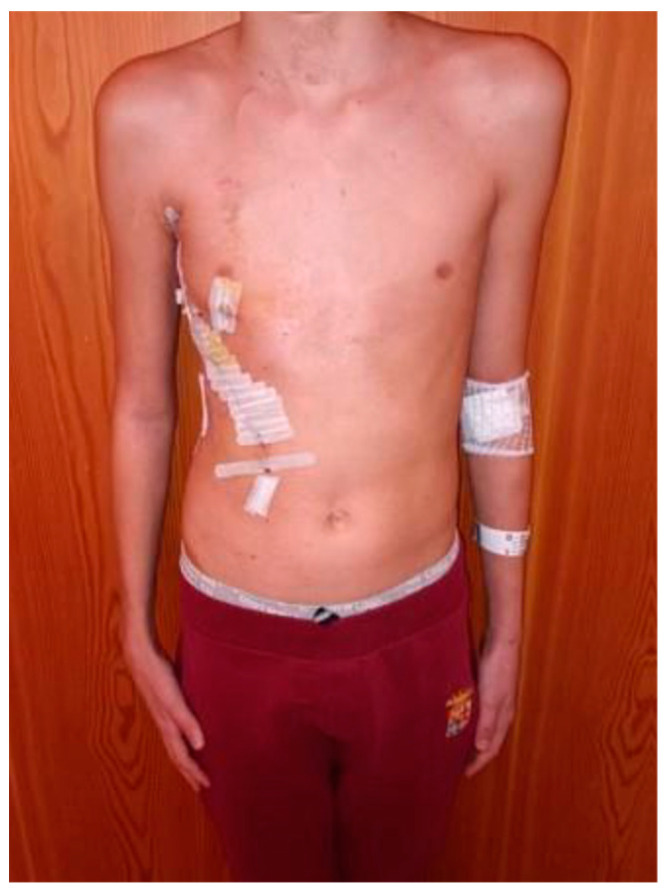
Aesthetics results.

No complications were encountered during surgery, and an aesthetically pleasing result was obtained (Figure 17).

The surgery was followed by four cycles of maintenance chemotherapy, and consolidation with Bu-Mel HDT plus autologous stem transplantation. An MRI performed one month after surgery did not record morphological or signal changes suspicious of signs of disease recurrence in residual tissues.

## 9. Conclusions

Our study confirms primary chest wall Ewing sarcoma as a rare and aggressive disease needing a multidisciplinary approach to achieve the best results in terms of OS and EFS. Our data, although based on a limited number of patients, agree with data reported in literature. In our experience, in fact, we have found significant benefits in performing pre-operative CT followed by surgery: induction chemotherapy allows a reduction of the tumor’s size and negative margins after surgical resection. The impact of the positive margins on OS and EFS could not be observed because our series reported a wide resection with clear margin (R0) in 100% of patients. Our series confirms metastases as the main negative prognostic factor. In regard to surgical reconstruction, biological materials were mainly used, which confirmed their benefit: they do not interfere with chest wall growth, and they do not act as a foreign body material, minimizing the risk of infection and mechanical stress to the chest wall during the physiological growth. Unfortunately, the lack of large series and prospective studies is a big limit in understanding the best therapeutic approach and the impact of prognostic factors in primary chest wall Ewing’s sarcoma.

## Figures and Tables

**Table 1:** Clinical data (age, sex; volume; CHT = chemotherapy; RT = radiotherapy; S = surgery, rib involvement, recurrence; metastasis). Y = yes; N = no.

Patient No.	Age (Year)	Sex	Primary Tumor: Involved Rib/Side	Pulmonary Involvement	Initial Tumor Volume (cm^3^)	Mtx (Ab Initio)	Enneking Class.	Treatment	Recurrence: Local (L) or Metastasis (M)
1	44	M	11th-12th/R	N	180	N	IIB	CHT+S	M
2	27	M	6th/R	N	84	N	IIA	CHT+S	N
3	10	M	11th-12th/R	Y	36	N	IIB	CHT+S	L
4	8	F	8th/R	N	180	N	IIB	CHT+S+RT	N
5	38	M	5th/R	N	41	N	IIB	CHT+S+RT	N
6	23	M	7th/R	N	72	N	IIA	CHT+S	L
7	18	M	3rd/R	N	180	N	IIA	CHT+S	-
8	39	F	3rd-4th-5th/R	N	ND	N	IIB	CHT+S+RT	M
9	15	F	6th-7th/L	N	153	N	IIA	CHT+S+CHT	N
10	61	F	3rd-4th/R	Y	11	N	IIB	CHT+S	L
11	19	F	3rd-4th/L	Y	6930	N	IIB	CHT+S+CHT	L, M
12	14	M	7th-8th-9th/R	N	560	N	IIA	CHT+S	N
13	19	M	3rd-4th/L	Y	52	N	IIB	CHT+S	N
14	20	F	1st-2nd-3rd/R	Y	243	N	IIB	CHT+S+CHT	L, M
15	2	F	9th/L	N	8	N	IIB	CHT+S+CHT	N

**Table 2:** Extent of resection (a) and reconstruction (b). (PTFE = expanded polytetrafluoroethylene).

**(a)**	
**Resection**	**n (%)**
Ribs	15 (100)
Lung wedge	3 (20)
Pneumonectomy	1 (6.6)
Diaphragm	2 (13)
**(b)**	
**Reconstruction**	**N (%)**
PTFE	4 (26.6)
PACLIDEM	11 (73.3)
Muscular flap	10 (66.6)
Titanium bars	6 (40)

**Table 3:** Operative and post-operative data.

Data Value	Mean ± SD or n (%)
Operative time	310 ± 120 (120–540) min
ICU stay	1.5 ± 0.6 (1–9) days
Hospital stay	7.8 ± 1.9 (5–11) days
Complications Atrial fibrillation Flap or wound infection Sieroma Anemia	5 (33.3) 1 (6.6) 1 (6.6) 2 (13.3) 1 (6.6)
Wild resection (type 1 margin)	13/15 (84.6%)

## Data Availability

All relevant data are within the manuscript.
